# The Brazilian breast cancer patient navigation program: impact on waiting times and treatment costs

**DOI:** 10.1093/heapol/czag105

**Published:** 2026-08-18

**Authors:** Alana Ramos da Silva, Fernando Antonio Slaibe Postali

**Affiliations:** Department of Economics, School of Economics, Business, Accounting and Actuarial Science, University of São Paulo (FEA/USP), Av. Prof. Luciano Gualberto, 908, Cidade Universitária, São Paulo, SP 05508-010, Brazil; Department of Economics, School of Economics, Business, Accounting and Actuarial Science, University of São Paulo (FEA/USP), Av. Prof. Luciano Gualberto, 908, Cidade Universitária, São Paulo, SP 05508-010, Brazil

**Keywords:** breast cancer, patient navigation, waiting time, healthcare costs, care coordination, synthetic difference-in-differences

## Abstract

Delays in cancer diagnosis and treatment pose significant challenges to the efficiency and equity of universal health systems. Patient navigation programs have been introduced to improve care coordination and facilitate timely access to treatment; however, evidence on their cost implications remains limited, particularly in middle-income countries. This study evaluates the impact of Brazil’s National Breast Cancer Patient Navigation Program on waiting times and treatment costs within the Unified Health System (Sistema Único de Saúde). Exploiting the nationwide implementation of the program in January 2023, we use administrative microdata covering January 2021 to June 2024. Gynecological cancers are used as a comparison group because they affect the same population and rely on similar hospital resources. Causal effects are estimated using the synthetic difference-in-differences approach. The results indicate that patient navigation reduced waiting time between diagnosis and radiotherapy initiation by ∼10 days relative to the comparison group, while effects on surgery and chemotherapy were limited. At the same time, radiotherapy costs increased by about 16.5%, suggesting a short-term expansion in service utilization reflecting previously unmet demand. The effects are heterogeneous across regions, with larger improvements observed in more developed areas. Overall, the findings indicate that patient navigation can improve access to breast cancer treatment but that its effectiveness depends on the availability of healthcare resources.

Key messages

- Patient navigation programs (PNPs) have emerged as a policy strategy to improve care coordination and reduce barriers to timely treatment, increasing access to specialized oncology services and enhancing health system responsiveness.
- Evidence on their impacts on healthcare costs and service utilization in middle-income countries remains limited.
- In Brazil, the breast cancer PNP significantly reduced the time between diagnosis and the initiation of radiotherapy by ∼10 days and was associated with a 16.5% increase in costs compared with the control group of patients with gynecological cancers.
- More substantial improvements were observed in more developed areas with greater availability of oncology services, indicating the need for strategies to enhance equitable access to specialized breast cancer treatment.

## Introduction

Breast cancer is the leading cause of cancer-related mortality among women in Brazil. In 2022, ∼19 000 women died from the disease, and about 73 000 new cases are expected in 2025 (National Cancer Institute—INCA 2024). Survival prospects critically depend on early diagnosis and the timely initiation of treatment (Silva 2018). Delays substantially reduce treatment effectiveness, increase healthcare costs, and generate significant losses in social welfare (Kaarboe and Carlsen 2014, Matias et al. 2025).

Brazil operates a universal public health system, the Unified Health System (Sistema Único de Saúde—SUS), which guarantees free access to cancer screening, diagnosis, and treatment. Despite national guidelines for breast cancer care, a lack of coordination contributes to pronounced inequalities in access across income groups, racial categories, educational levels, and geographic regions (Srinath et al. 2023, Lemos et al. 2024, Antonini et al. 2025, Freitas-Júnior et al. 2025, Silva and Scorzafave 2025). Although services are free at the point of use, demand frequently exceeds short-term treatment capacity (Kaarboe and Carlsen 2014, Carlsen et al. 2025). Consequently, a substantial share of breast cancer cases in Brazil are diagnosed at advanced stages, when therapeutic options are more limited and more costly (Silva 2018).

Delays in diagnosis and treatment pose a major challenge for the efficiency of the Brazilian public health system. Empirical evidence consistently documents persistent failures in timely access to breast cancer care in Brazil (Bretas et al. 2021, Sobral et al. 2022, Migowski et al. 2025). In 2022, more than half of breast cancer patients treated in the SUS initiated treatment after the legally mandated deadline of 60 days following diagnosis (INCA 2022). Long waiting times are commonly interpreted as indicators of poor service quality (Monstad et al. 2014) and have important implications for public health expenditures. As treatment is delayed and the disease progresses, costs increase substantially (Oncology Observatory 2023). Moreover, the economic burden of cancer extends beyond direct medical expenditures and also includes indirect costs (Cong et al. 2022). Delays also impose substantial physical and psychological stress on patients and their families.

A growing body of literature identifies fragmented care pathways and weak coordination among health professionals as central drivers of inefficiency in universal health systems (Kaarboe and Carlsen 2014, Lombardo and Popim 2020, Simonsen et al. 2020, Chan et al. 2023). In response to these coordination failures, patient navigation programs (PNPs) have been implemented in several high-income countries as organizational innovations designed to help patients navigate complex healthcare systems (Louart et al. 2021, Budde et al. 2022). These programs aim to reduce information barriers, coordinate care, and facilitate timely access to treatment (Louart et al. 2021, Matias et al. 2025).

Patient navigators—typically nurses or social workers—assist patients throughout the care pathway by facilitating referrals, coordinating appointments, providing written documentation of intended treatment types, locations, and dates, reducing informational barriers, and supporting timely access to diagnostic and treatment services (Louart et al. 2021, Chan et al. 2023, World Health Organization—WHO 2024). By addressing administrative bottlenecks and improving care coordination, navigation programs may shorten the interval between diagnosis and treatment initiation, particularly in health systems characterized by fragmented service delivery. Although patient navigation is expected to improve access across all major treatment modalities, its effects may be particularly relevant for services that depend on complex referral pathways and scarce specialized resources (Chen et al. 2024). In Brazil, access to treatments such as radiotherapy often involves multiple administrative steps and capacity constraints, making care coordination especially important for reducing delays (Vieira et al. 2023).

Care coordination can affect both service utilization and healthcare costs, particularly when previously unmet needs are addressed (Ress and Wild 2023). By reducing barriers to access and facilitating patient flow through the healthcare system, navigation programs may increase the utilization of diagnostic and treatment services among patients who previously experienced delays or difficulties accessing care. As a result, healthcare expenditures may increase in the short run as suppressed demand is addressed, even if earlier treatment ultimately generates health and economic benefits over time (Aguiar-Ibáñez et al. 2025, Kong et al. 2025). Empirical findings remain mixed, and existing evidence has not conclusively demonstrated that integrated care models consistently reduce costs while improving health outcomes (Goldzahl et al. 2022, Lugo-Palacios et al. 2023). From the patient perspective, however, studies from high-income countries suggest that patient navigation improves satisfaction, quality of life, access to diagnostic services, and reduces waiting times (Rodrigues et al. 2021, Chan et al. 2023, Chen et al. 2024). Rigorous evidence on the effectiveness of patient navigation in middle-income countries and universal public health systems remains scarce (Louart et al. 2021).

In 2022, Brazil introduced the National Breast Cancer Patient Navigation Program through Law No. 14.450, formally integrating patient navigation into the SUS. The program aims to provide individualized and timely support to women diagnosed with breast cancer, with the explicit objective of reducing delays and improving care coordination, thereby lowering treatment costs and improving health outcomes. Despite its potential relevance, no large-scale causal evaluation of this policy has been conducted to date.

This study evaluates the impact of Brazil’s National Breast Cancer Patient Navigation Program on waiting times and federal treatment costs using nationwide administrative microdata from the SUS. Exploiting the program’s implementation, we apply synthetic difference-in-differences (SDID) method to estimate its causal effects. Gynecological cancers are used as a comparison group because they affect the same population and rely on similar oncology infrastructure and hospital resources (Cerqueira-Silva et al. 2025).

We hypothesize that improved care coordination reduces waiting times and leads, in the short run, to increased service utilization as previously unmet demand is addressed. As a result, total treatment costs may rise as patients who previously faced barriers to access receive timely care. To our knowledge, this study provides the first large-scale causal evaluation of a national patient navigation policy within Brazil’s universal healthcare system. Our findings contribute to the literature in three ways. First, we provide causal evidence on the effects of patient navigation in a middle-income country. Second, we examine how care coordination affects both access to treatment and healthcare costs. Third, we offer new evidence on policies aimed at improving efficiency and reducing inequalities in universal healthcare systems.

## Background on the national breast cancer patient navigation program

Breast cancer care within the Brazilian Unified Health System (SUS) can be summarized into three sequential intervals: the patient interval, the diagnostic interval, and the treatment interval (Duggan et al. 2020). Delays at any stage can worsen prognosis and increase treatment costs, particularly when diagnosis occurs at more advanced stages.

Biennial screening is recommended for asymptomatic women aged 50–69 and for women at high risk due to genetic factors or family history, while symptomatic women are referred directly for diagnostic evaluation (Migowski et al. 2018). Mammography does not provide a definitive diagnosis but indicates the likelihood of malignancy, guiding further diagnostic procedures such as biopsy. Confirmation of malignancy marks the transition from screening to diagnostic care, followed by disease staging and treatment selection (Duggan et al. 2020). Treatment options depend on disease severity and may include surgery, radiotherapy, systemic therapies, or palliative care. Advanced-stage breast cancer is associated with significantly higher treatment intensity and costs, making delays in diagnosis and treatment initiation particularly detrimental to both patient outcomes and health system efficiency (Migowski et al. 2025).

In response to coordination failures along this care pathway, Brazil has enacted several legal frameworks aimed at reducing delays in cancer care. These include legislation establishing a maximum period of 60 days between diagnosis and treatment initiation (Law No. 12.732/2012) and a 30-day limit for diagnostic confirmation (Law No. 13.896/2019). In 2022, Law No. 14.450 established the National Breast Cancer Patient Navigation Program within the SUS.

In Brazil, patient navigation has historically lacked a standardized model, with initiatives implemented in a fragmented and localized manner (Lombardo and Popim 2020, Rodrigues et al. 2021). The National Breast Cancer Patient Navigation Program aims to improve care coordination for women with suspected or confirmed breast cancer by offering individualized support throughout the entire care pathway. Specifically, the program seeks to: (i) promote compliance with legally established deadlines for diagnosis and treatment initiation; (ii) support the training of health professionals involved in cancer care; (iii) assist patients in navigating health services and understanding their rights within the health system; (iv) reduce inefficiencies and treatment-related costs; and (v) provide continuous, individualized care coordination for women with suspected or confirmed breast cancer (Brazil 2022).

By addressing informational frictions and administrative bottlenecks, the program is expected to reduce delays between diagnosis and treatment initiation and, consequently, lower treatment costs in the long-term. The program came into effect in 2022 and was later incorporated into the National Cancer Patient Navigation Program through Law No. 14.758 of December 2023, which extended navigation services to all cancer types starting in June 2024. Although the legal framework establishes nationwide coverage, effective implementation may vary across municipalities and over time due to differences in local health system capacity.

## Methods

### Study design and data sources

To evaluate the effects of the Breast Cancer PNP on waiting times and treatment costs, we use four publicly available datasets from three administrative systems maintained by the Department of Informatics of the Unified Health System (DATASUS). We examine two primary outcomes—waiting times and federal treatment transfers—across three cancer treatment modalities: oncological surgery, radiotherapy, and chemotherapy. The analysis is restricted to cancers affecting women.

For each outcome, we construct panels according to each treatment modality (codes is publicly available). Panels are constructed at the patient level and aggregated monthly by cancer type, which allows us to examine changes before and after the implementation of the PNP in January 2023. This structure enables the application of a SDID design to evaluate the national introduction of the program.

Microdata on oncology procedure costs were extracted using the *microdatasus* package in R, following the procedures described by Saldanha et al. (2023). Data on waiting times were obtained from the Oncology Panel, which is made available by DATASUS through the TabNet platform as pre-processed and consolidated files. The study period covers cancer diagnoses recorded between January 2021 and June 2024, totaling 42 months.

### Outcomes (waiting time and costs)

Waiting-time outcomes are constructed using data from the oncology panel, which is derived from the outpatient information system (SIA-SUS). The oncology panel was launched in 2013 to monitor compliance with Federal Law No. 12.732/2012 (“60-day law”)—which mandates treatment initiation within 60 days after diagnosis. This dataset reports the time interval between diagnosis and first treatment for the three treatment modalities, along with patient-level information on age, sex, municipality of residence, International Classification of Diseases (ICD-10) codes, and cancer stage for cases treated with chemotherapy and radiotherapy.

All cost measures reflect SUS reimbursement values. SUS providers are reimbursed according to a fixed fee schedule for each procedure, regardless of the actual cost of service provision. These reimbursement amounts are administratively set and have not been updated since 2013. Therefore, total cost measures are mechanically proportional to the number of procedures, and should be interpreted as a monetary representation of service volume rather than an independent outcome. Information on treatment costs is obtained from two administrative systems. Data on surgical procedures are drawn from the hospital information system (SIH-SUS), which records hospital admission authorizations (Autorizações de Internação Hospitalar). These records include information on patient characteristics (e.g. sex, municipality of residence, and ICD-10 code), hospital location, and the monetary value reimbursed for each inpatient procedure.

Data on chemotherapy and radiotherapy are obtained from the outpatient information system (SIA-SUS), one of the main administrative databases of the Brazilian public health system. The SIA-SUS records outpatient procedures, including chemotherapy and radiotherapy sessions. These procedures are authorized through outpatient procedure authorizations (Autorizações de Procedimentos Ambulatoriais—APAC). In this study, we use data from the high-complexity APAC categories AQ (chemotherapy) and AR (radiotherapy).

Although patients are not explicitly identified, APAC records include an identification mask and information on age, sex, race/skin color, ICD-10 code, date of diagnosis, cancer stage, and the monetary value reimbursed for each authorized procedure. Monetary values were adjusted for inflation using the Brazilian Consumer Price Index (IPCA), published by the Brazilian Institute of Geography and Statistics (IBGE). All costs are expressed in constant June 2024 Brazilian reais. The inflation adjustment was applied to the monthly aggregated cost series prior to estimation.

### Covariates

The patient-level panel structure guides the selection of covariates. Because data come from multiple administrative systems, available covariates vary across sources. Cost estimations for chemotherapy and radiotherapy include age, race (White or Black), and cancer stage (I–IV). For surgical cost estimations, we include age and race. In the waiting-time regressions, we control for age and cancer stage, except for surgical treatments, for which information on cancer stage is not available.

### Descriptive analyses and pre-trends

A key challenge of the quasi-experimental design is identifying a credible counterfactual for breast cancer. To address this issue, we compare outcomes across cancer types to identify a suitable control group. Table 1 reports average waiting times across treatment modalities before and after the implementation of the program.

**Table 1:** Descriptive statistics (I).

Cancer	Mean waiting time	Before the navigation program	After the navigation program
All	88.8	98.2	76.8
Surgery	18.9	21.8	15.1
Chemotherapy	100.3	109.0	89.6
Radiotherapy	175.2	199.6	142.1
Breast	106.4	113.0	98.1
Surgery	56.6	62.4	49.0
Chemotherapy	109.3	116.7	100.1
Radiotherapy	182.2	191.0	173.1
Gynecological	74.4	78.3	69.3
Surgery	15.9	18.0	13.0
Chemotherapy	102.4	110.9	91.9
Radiotherapy	132.2	135.6	127.6

This table presents descriptive statistics for cancer diagnosis data between January 2021 and June 2024, before and after the implementation of the Navigation Program in January 2023. The data comes from the Oncology Panel/DATASUS. “All” indicates all cancers among female patients (ICD-10 C00-C97); “Breast” indicates the averages for breast cancer only (C50), and “Gynecological” shows the means for female cancers only, such as cervical and vaginal cancer (C51-C58).

The first column presents statistics for all malignant neoplasms (ICD-10 C00–C97), including cancers affecting both men and women. The second column reports outcomes for breast cancer (ICD-10 C50), while the third column presents averages for gynecological cancers (ICD-10 C51–C58). This group includes cancers of the vulva (C51), vagina (C52), cervix uteri (C53), corpus uteri (C54), uterus not otherwise specified (C55), ovary (C56), other and unspecified female genital organs (C57), and placenta (C58).

Table 1 shows that breast cancer is associated with longer waiting times than other cancers, while chemotherapy waiting times are similar between breast and gynecological cancers. Waiting times decline across modalities over time. In addition, radiotherapy has the longest average waiting time, reflecting greater technological complexity and supply constraints.

Descriptive statistics for treatment mean costs are presented in Table 2. Radiotherapy has the highest per-patient cost and represents the most expensive treatment modality for breast cancer.

**Table 2:** Descriptive statistics (II).

Cancer	*N* (procedures)|mean cost (BRL)	Before the navigation program	After the navigation program
All			
Surgery	1 309 247 (2566.41)	700 247 (2523.90)	609 000 (2615.29)
Chemotherapy	7 391 665 (504.53)	3 843 995 (498.13)	3 547 670 (511.46)
Radiotherapy	226 735 (4965.67)	117 053 (4944.51)	109 682 (4988.26)
Breast			
Surgery	277 213 (2418.98)	145 874 (2357.64)	131 339 (2487.11)
Chemotherapy	6 308 510 (352.56)	3 290 721 (350.66)	3 017 789 (354.63)
Radiotherapy	103 187 (5969.63)	51 453 (5975.83)	51 734 (5963.46)
Gynecological			
Surgery	196 137 (2754.51)	106 254 (2724.48)	89 883 (2790.00)
Chemotherapy	311 791 (1038.50)	160 425 (1047.01)	151 366 (1029.48)
Radiotherapy	75 371 (4405.80)	40 384 (4405.37)	34 987 (4406.31)

The data come from SIA/DATASUS. “All” indicates all cancers among female patients (ICD-10 C00–C97); “Breast” indicates breast cancer only (C50); and “Gynecological” refers to female cancers such as cervical and vaginal cancer (C51–C58). Mean costs in Brazilian reais (BRL) are reported in parentheses. Pre-intervention period: 24 months; Post-intervention period: 18 months.

Based on these patterns, we trace the time trajectories of average waiting times and the natural logarithm of federal treatment costs for breast cancer (blue lines) and gynecological cancers (gray lines), with the intervention occurring in January 2023 (Law No. 14.450 establishing the PNP for women diagnosed with breast cancer was enacted in September 2022. However, the empirical analysis considers January 2023 as the beginning of the intervention period, as this allowed time for health services to adapt to the new legal requirements and implement the navigation procedures in practice.). Figure 1 illustrates the evolution of waiting times over the study period.

**Figure 1:**
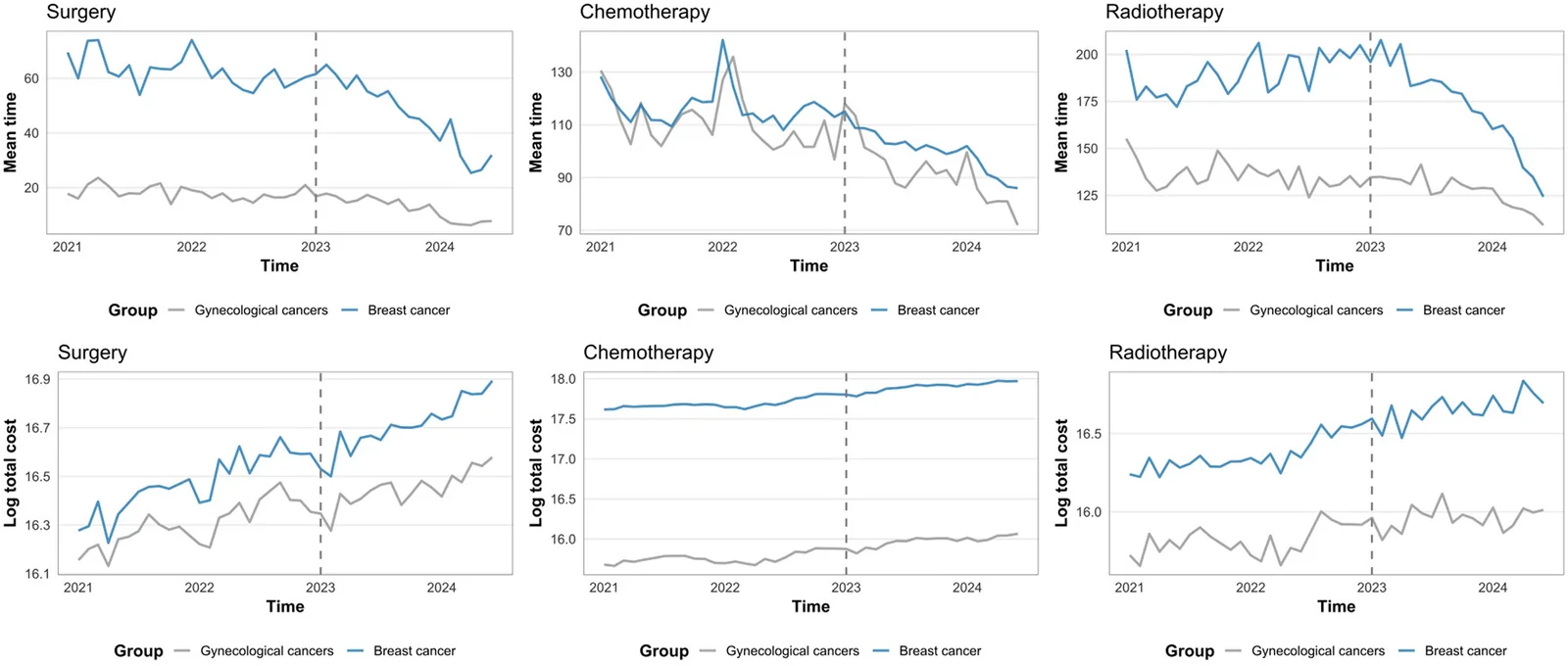
Trends in treatment and control groups. Trends in mean waiting time and natural logarithm of total treatment cost for breast cancer in the modalities of surgery, chemotherapy, and radiotherapy from January 2021 to June 2024 (42 months of analysis). The navigation program intervention occurs in January 2023. The blue trajectory represents breast cancer, while the gray trajectories represent gynecological cancers.

Visual inspection suggests limited plausibility of the parallel trends assumption for surgery and chemotherapy, particularly for waiting times, as the treated and comparison groups display distinct pre-intervention trajectories. In contrast, pre-intervention trends for radiotherapy appear more closely aligned across groups, especially for treatment costs, supporting the credibility of the identifying assumptions in this case. These patterns motivate the use of the SDID estimator as the primary identification strategy.

### Empirical strategy

Descriptive analyses suggest that the SDID estimator is the most appropriate method to estimate the effects of the PNP on waiting times and treatment costs. The intervention was implemented nationwide in January 2023 and initially targeted breast cancer patients. Although gynecological cancers differ clinically from breast cancer, SDID adjusts for pre-intervention differences in levels and trends.

To construct the control group, we adapted the strategy of Cerqueira-Silva et al. (2025) and selected gynecological cancers (ICD-10 C51–C58) as the control group to breast cancer (ICD-10 C50). These conditions affect the same population and are treated within the same hospital units, relying on similar oncology infrastructure and resources within the Brazilian Unified Health System (SUS). As a result, both groups are exposed to similar institutional constraints, including the availability of diagnostic services and treatment capacity. In addition, both breast and cervical cancer (ICD-10 C53) are priority targets of national screening and treatment policies in Brazil, with mammography and Pap smear serving as the main screening methods. Importantly, the PNP initially targeted breast cancer and did not include other female cancers, which allows gynecological cancers to serve as a comparison group to assess whether changes in waiting times and treatment costs can be attributed to the program.

Visual inspection suggests that pre-intervention trends differ between the two cancer groups. Furthermore, event-study analysis for difference-in-differences (DID) provides no evidence to support the parallel trends assumption, as shown in Supplementary Figure S1. These findings justify the use of the SDID approach as a more suitable alternative to address this limitation.

Let $T_{t}^{k}$ denote the health outcome for women diagnosed with breast cancer, and $C_{t}^{k}$ the corresponding outcome for women diagnosed with gynecological cancer (the control group). The superscript *s* indicates two possible situations: participation in the Breast Cancer PNP, defined as women diagnosed with breast cancer from January 2023 onward, and non-participation (*N*). The subscript t refers to time, where the pre-intervention period (t=0) covers January 2021–December 2022, and the post-intervention period (t=1) covers January 2023 to June 2024. The effect of the PNP can therefore be expressed as:


(1) $$\delta_{\mathrm{PNP}}=(T_{1}^{\mathrm{PNP}}-T_{0}^{\mathrm{PNP}})-(C_{1}^{N}-C_{0}^{N})$$

Equation (1) represents the change in outcomes for the treated group relative to the change observed in the control group. Under the parallel trends assumption, δ identifies the causal effect of the program. To strengthen the counterfactual design, we employ the SDID estimator proposed by Arkhangelsky *et al*. (2021) and computationally implemented by Clarke *et al*. (2024). SDID combines features of DID and synthetic control methods and is defined as:


(2) $$({\hat{\beta }}^{\mathrm{SDID}},\;\hat{\mu },\;\hat{\gamma },\;\hat{\tau })=\arg\;\underset{\beta ,\mu ,\gamma ,\tau }{\min}\;\left\{\overset{n}{\underset{s=1}{\sum }}\;\overset{t}{\underset{t=1}{\sum }}\;{\left(Y_{st}-\mu -\gamma_{s}-\tau_{t}-PN_{\mathrm{st}}\beta \right)}^{2}\;{\hat{\omega }}_{s}^{\mathrm{SDID}}\;{\hat{\lambda }}_{t}^{\mathrm{SDID}}\right\}$$

where ${\hat{\beta }}^{\mathrm{SDID}}$ represents the average treatment effect on the treated (ATT) for women with breast cancer. The estimator is derived from a two-way fixed effects model with optimally chosen unit weights ${\hat{\omega }}_{s}^{\mathrm{SDID}}$ and time weights ${\hat{\lambda }}_{t}^{\mathrm{SDID}}$. Unlike standard DID, SDID relaxes the strict parallel trends assumption; instead, it adjusts for pre-treatment differences by selecting a weighted combination of control units that best matches the treated group’s pre-intervention trajectory. Time weights are also optimally chosen to mitigate bias from unobserved shocks and improve estimation accuracy (Saffer et al. 2025).

We estimate Equation (2) separately for the three treatment modalities. SDID estimates are obtained using the *synthdid* package in RStudio. Statistical inference relies on placebo-based standard errors from 1000 random reassignments of treatment status. As discussed in Arkhangelsky et al. (2021) and Clarke et al. (2024), this procedure is suitable when only a single treated unit is available. Moreover, SDID requires a balanced panel; therefore, given the nature of our data, outcomes are aggregated at the monthly level by cancer type. Cancer types that do not appear in all months of the analysis are excluded.

To assess the plausibility of the parallel trends assumption prior to the intervention, we construct event-study plots. For SDID, following Saffer et al. (2025), we plot the conditional difference between the treated group and its synthetic control over time. Since the average pre-intervention difference is subtracted from each period’s difference, the reference category corresponds to the entire pre-policy period. We report 95% confidence intervals based on bootstrap procedures.

As a sensitivity analysis, we estimate interrupted time series (ITS) regression models following the approach of Linden and Arbor (2015), separately for breast and gynecological cancers, using January 2023 as the intervention date. The absence of statistically significant changes among gynecological cancers serves as a robustness check, as these cancers were not targeted by the PNP. In addition, we assess potential compositional changes in patient severity among chemotherapy and radiotherapy patients by estimating SDID models for the share of advanced-stage (III–IV) cases. Finally, we exclude states that received linear accelerators under the radiotherapy expansion plan (PER-SUS) during the study period to mitigate potential confounding from concurrent expansions in radiotherapy infrastructure that could independently affect service capacity and waiting times.

## Results

### Main results on the impact of PNP

Table 3 presents the main estimates of the impact of the PNP on waiting times and treatment costs across three treatment modalities: surgery, chemotherapy, and radiotherapy. SDID estimates are interpreted as average monthly effects relative to the synthetic control group, rather than differences in cumulative totals across pre- and post-intervention periods. Overall, the results indicate that the program had the largest and most consistent effects on radiotherapy, reducing waiting times and increasing treatment costs, while effects for surgery and chemotherapy are weaker or statistically insignificant.

**Table 3:** Estimates of the impact of the navigation program on mean waiting time and total cost for different cancer treatments.

	Panel A: surgery		Panel B: chemotherapy		Panel C: radiotherapy	
	DID	SDID	DID	SDID	DID	SDID
Mean time	−7.561***	−10.586	2.530	−1.697	−14.569***	−10.198***
SE	(2.572)	(7.490)	(1.581)	(2.682)	(3.165)	(0.266)
*P*-value	.003	.158	.109	.527	0	0
*N*	67 026	32	160 012	28	36 978	30
Total cost	−0.007	0.052	0.008	−0.008	0.003	0.153***
SE	(0.011)	(0.156)	(0.012)	(0.030)	(0.002)	(0.021)
*P*-value	.482	.738	.479	.796	.204	0
*N*	473 298	36	6 547 495	6	175 730	36

The table reports DID estimates with cluster-robust standard errors and SDID estimates with placebo-based standard errors obtained from 1000 random placebo replications, along with *P*-values and sample sizes. The DID models use fixed effects of municipality and time and control for age, race/ethnicity, and cancer stage.

^***^ *P* < .01; **P < .05; *P < .10.

Supplementary Figure S2 displays the trajectories of the treated group (breast cancer) and the corresponding synthetic control constructed using the SDID weights. Figure 2 presents the event-study plots based on the SDID models. These plots indicate a good pre-treatment fit between the treated and synthetic control groups across outcomes and treatment modalities. Supplementary Table S1 reports the unit weights used to construct the synthetic control group. Cervical and uterine body cancers account for the largest weights in the SDID estimation, indicating that these cancer types contribute most to matching the pre-intervention trajectory of breast cancer outcomes.

**Figure 2:**
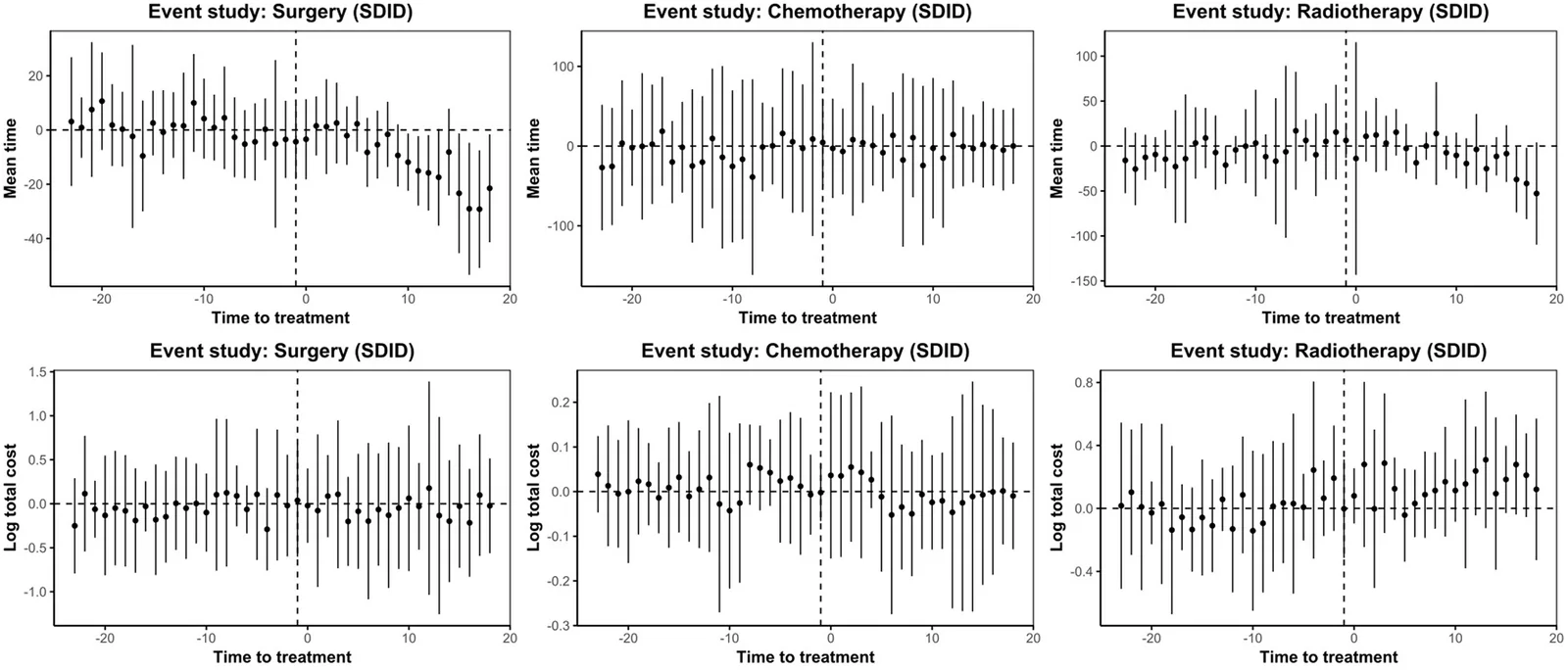
Event study. This figure presents the event-study estimates from the SDID analysis reported in Table 3. The points represent the estimated treatment effects, and the error bars indicate 95% confidence intervals obtained using the bootstrap method.

We place greater emphasis on the SDID estimates, as this estimator relaxes the strict parallel trends assumption required by conventional DID. For surgery (Panel A of Table 3), we find a statistically significant reduction in mean waiting time in the DID specification. Although the SDID estimates are similar in magnitude, they are not statistically significant. For chemotherapy (Panel B), we do not find statistically significant effects of PNP on either waiting times or costs using either estimator.

In contrast, for radiotherapy (Panel C), we observe statistically significant effects of PNP on both mean waiting time and total costs. The estimated average treatment effects on the treated (ATT) indicate a reduction in mean waiting time of 14.6 days in the DID model and 10.2 days in the SDID model. In terms of costs, the SDID estimates indicate a statistically significant increase of ∼16.5%, computed as $\left[(e^{0.153}-1)\times 100\right]$, based on the log specification. This is the main estimate, as it is scale-invariant and reflects the relative effect of the program compared with the synthetic control. This effect should be interpreted as a model-based relative change rather than a direct change in observed expenditures. A causal interpretation of these estimates is supported by the balanced pre-intervention trends observed in the SDID event-study plots (Fig. 2).

Although the SDID estimates for radiotherapy indicate a statistically significant average post-treatment effect, the corresponding event-study plots display relatively imprecise period-specific estimates. These plots nonetheless suggest similar pre-treatment dynamics between breast and gynecological cancers, supporting the plausibility of the identifying assumptions. The imprecision of the dynamic estimates likely reflects the limited aggregate sample size underlying the SDID analysis. This apparent discrepancy arises because the SDID estimator aggregates treatment effects over time using optimal weights, whereas the event-study estimates dynamic effects separately for each period. In the case of radiotherapy, treatment effects appear to be modest but persistent throughout the post-intervention period, resulting in a statistically significant average effect despite wide confidence intervals at the individual period level.

### Robustness checks

Excluding individual-level covariates does not materially affect the main SDID estimates. In particular, the estimated effects for radiotherapy remain statistically significant and similar in magnitude to the baseline specification. Moreover, the SDID estimates generally exhibit the same direction as the DID estimates reported in Table 3, reinforcing the consistency of the results across estimators.

As an additional robustness check, we estimate ITS models separately for breast cancer and gynecological cancers using January 2023 as the intervention date (Supplementary Tables S2 and S3). For breast cancer, the ITS models indicate statistically significant post-intervention reductions in waiting times across all treatment modalities, with the largest effect observed for radiotherapy. The estimated changes in trends correspond to monthly reductions of 1.8 days for surgery, 1.2 days for chemotherapy, and 5.0 days for radiotherapy. In contrast, we do not observe statistically significant changes in treatment costs over time.

For gynecological cancers, which were not targeted by the PNP, we do not observe statistically significant changes in either levels or trends. The absence of similar effects in this comparison group provides additional support that the observed reductions in waiting times are concentrated among breast cancer patients exposed to the intervention.

We further assess whether changes in patient severity may have influenced the results by estimating SDID models for the share of advanced-stage (III–IV) cases among chemotherapy and radiotherapy patients (Supplementary Table S4). The results indicate heterogeneous compositional changes across treatment modalities. The proportion of advanced-stage cases decreased by 6.4 percentage points among radiotherapy patients but increased by 1.6 percentage points among chemotherapy patients. These findings suggest that some changes in case composition may have occurred following program implementation, particularly in radiotherapy services. This is consistent with the possibility that improved coordination and earlier access may have facilitated treatment of patients at less advanced stages. At the same time, the heterogeneity across modalities indicates that compositional changes are not uniform across the system.

Finally, to address the possibility that the expansion of radiotherapy infrastructure through the Radiotherapy Expansion Plan (PER-SUS) may have influenced the results, we re-estimate the radiotherapy SDID models after excluding states that received new linear accelerators during the post-intervention period (Supplementary Tables S5a and S5b). The estimated effects remain statistically significant at the 5% level, with waiting times reduced by ∼7 days and radiotherapy costs increasing by 4.8%. Although the magnitudes are somewhat smaller than those obtained in the baseline specification, the direction and statistical significance of the effects are preserved, suggesting that the main findings are not driven solely by concurrent expansions in radiotherapy capacity.

Because cervical cancer (C53) received a large weight in the SDID estimation, we conducted an additional robustness check excluding this cancer from the donor pool (Supplementary Table S6). The estimated effects for radiotherapy remain robust across outcomes. For waiting times, the ATT remains statistically significant and similar in magnitude to the baseline specification (−9.2 versus −10.2 days). For radiotherapy costs, the estimated effect is also stable, with an increase of ∼13.2% compared with 16.5% in the baseline model. Overall, these results indicate that the main findings are not driven by the contribution of cervical cancer to the synthetic control.

### Heterogeneous effects by macroregion

Although the legal framework of the SUS establishes nationwide coverage, the effective implementation of PNP may vary across regions due to differences in local health system capacity. Brazil is divided into five macroregions that differ substantially in health system capacity and access to healthcare services. The North and Northeast regions face the most severe barriers to healthcare access (Silva and Postali 2025), whereas the South, Southeast, and Midwest regions—hereafter referred to as the Center-South—exhibit better health system performance, with the South and Southeast standing out in terms of access indicators.

Motivated by these disparities, we examine heterogeneity in the effects of PNP across macroregions. Results for the Center-South and the North-Northeast regions are reported in Tables 4 and 5, respectively.

**Table 4:** Heterogeneous effects by macroregion (I).

	South central					
	Surgery		Chemotherapy		Radiotherapy	
	DID	SDID	DID	SDID	DID	SDID
Mean time	−9.941***	−15.856*	−0.073	−1.654	−14.412***	−9.978***
SE	(2.705)	(9.228)	(1.851)	(3.646)	(3.818)	(2.065)
*P*-value	0	.086	.968	.650	0	0
*N*	50 534	24	110 782	36	23 544	35
Total cost	−0.002	0.071	0.020	0.004	0.004	0.192***
SE	(0.014)	(0.105)	(0.014)	(0.029)	(0.006)	(0.045)
*P*-value	.888	.496	.159	.889	.512	0
*N*	334 970	36	4 833 840	12	120 520	36

The table presents the DID and SDID estimates for each outcome, robust, and placebo-controlled standard errors, *P*-values, and sample sizes. The DID models use individual-level data that control for age, cancer stage, and region of residence.

^***^ *P* < .01; **P < .05; *P < .10.

**Table 5:** Heterogeneous effects by macro-region (II).

	North and northeast					
	Surgery		Chemotherapy		Radiotherapy	
	DID	SDID	DID	SDID	DID	SDID
Mean time	2.021	−1.011	8.672***	6.440*	−4.637	0.176**
SE	(4.215)	(1.536)	(2.729)	(3.675)	(6.489)	(0.087)
*P*-value	.631	.510	.001	.079	.475	.044
*N*	16 492	42	49 979	25	13 482	25
Total cost	−0.023	−0.050	−0.024*	−0.035	0.001	0.082***
SE	(0.020)	(0.275)	(0.014)	(0.321)	(0.010)	(0.027)
*P*-value	.245	.855	.077	.914	.880	.003
*N*	138 328	24	1 713 655	15	55 210	30

The table presents DID estimates with standard errors per cluster and SDID estimates with standard errors per placebo, *P*-values, and sample sizes. The DID models use fixed effects of municipality and time and control for age, race/ethnicity, and cancer stage.

^***^ *P* < .01; **P < .05; *P < .10.

In the Center-South region, we find statistically significant reductions in waiting times for both surgery and radiotherapy in the DID and SDID models. Estimated effects indicate reductions of 9.9 and 15.8 days in surgical waiting times under the DID and SDID specifications, respectively. For radiotherapy, waiting times decrease by 14.4 days in the DID model and 10.0 days in the SDID model. In addition, radiotherapy costs increase by ∼21.2% $[(e^{0.192}-1)\cdot 100],$ according to the SDID estimates. No statistically significant effects are observed for chemotherapy.

In contrast, estimates for the North and Northeast regions indicate increases in average waiting times for chemotherapy. The DID and SDID models suggest increases of 8.7 and 6.4 days, respectively. For radiotherapy, the SDID estimates point to increases in both waiting times and costs, of ∼0.18% and 8.5%, respectively. No significant effects are detected for surgery. Given the relatively small sample size for the North region, we interpret these heterogeneous treatment effects with caution and view them as suggestive rather than conclusive.

## Discussion

Waiting times are a key dimension of health system responsiveness (Simonsen et al. 2020), and delays in the initiation of treatment are detrimental both to patient outcomes and to the efficiency of healthcare systems (Matias et al. 2025, Migowski et al. 2025). Integrated care models, such as PNPs, are widely recognized as mechanisms to improve access to and utilization of health services. However, evidence regarding their ability to reduce healthcare costs remains mixed and controversial (Goldzahl et al. 2022).

Our analysis contributes to this debate by exploiting the quasi-natural experiment generated by the implementation of a national patient navigation policy targeting breast cancer—one of the most prevalent cancers among women in Brazil. Gynecological cancers serve as the control group because they affect the same population and share similarities in diagnostic pathways and treatment strategies. By doing so, this study provides evidence that can inform future policies aimed at reconciling the goals of improving access to treatment while maintaining fiscal sustainability, ensuring improved health outcomes in middle-income countries (Apantaku et al. 2025).

We find that the breast cancer PNP reduced the waiting time between diagnosis and the initiation of radiotherapy by ∼10 days relative to the control group, while radiotherapy costs increased by about 16.5%. We do not observe comparable effects for surgery and chemotherapy. This pattern may reflect heterogeneity in treatment pathways and bottlenecks across modalities.

Patient navigation primarily targets coordination, referral, and scheduling barriers along the cancer care continuum. Radiotherapy may be particularly responsive to such interventions because it combines multi-step treatment pathways, requiring referral, planning, and repeated sessions, with reliance on specialized and geographically concentrated infrastructure (Viani et al. 2022, Chan et al. 2023, Chen et al. 2024, WHO 2024). In this context, recent capacity expansion in radiotherapy services may have further increased the relative importance of coordination frictions in determining access.

Moreover, the effects of the program are heterogeneous across Brazilian regions. In the more developed macroregions, waiting times for surgery and radiotherapy declined, while costs increased for radiotherapy. In comparison, in the North and Northeast regions—historically characterized by poorer health access indicators—we observe increases in waiting times for chemotherapy.

These findings support the hypothesis that patient navigation improves access to treatment but may increase healthcare expenditures in the short run. Previous studies suggest that earlier diagnosis and treatment may be associated with long-term societal and economic benefits through improved health outcomes, lower costs associated with advanced disease, and productivity gains (Aguiar-Ibáñez et al. 2025, Kong et al. 2025).

Nevertheless, some limitations must be acknowledged. First, our estimates depend on the quality and completeness of administrative data, and we cannot rule out errors or omissions in the records. Second, given the recent implementation of the policy, our estimates capture short-term effects and may not reflect longer-term adjustments in capacity and resource allocation. Third, the approval of a law does not necessarily imply its immediate or complete implementation. Therefore, favorable outcomes may coexist with shortcomings or delays in the implementation process, which could not be evaluated with the available data. Finally, although gynecological cancers were selected as a comparison group because they affect a similar population and rely on comparable oncology infrastructure, unobserved factors such as workforce shortages, service capacity constraints, and fluctuations in healthcare demand may have influenced outcomes in both groups. Therefore, residual confounding cannot be completely excluded.

Despite these limitations, this study adds to the literature by providing the first large-scale evaluation of a national breast cancer PNP within SUS. To the best of our knowledge, no prior impact evaluation of this policy has been conducted at the national level. Our findings, particularly the reduction in waiting times for radiotherapy, indicate that patient navigation has the potential to expand access to specialized cancer treatment. However, ensuring an equitable distribution of these gains across regions remains a critical challenge.

The observed reduction in radiotherapy waiting times is consistent with evidence from navigation programs in high-income countries, which generally find that navigators shorten the intervals between screening, diagnosis, and treatment initiation (Rodrigues et al. 2021, Chan et al. 2023, Chen et al. 2024). Pilot programs in several European countries also show that navigation improves care coordination for chronic diseases, especially during transitions across stages of care (Budde et al. 2022). More broadly, low- and middle-income countries have substantially fewer surgical facilities and more limited access to cancer treatment compared with high-income countries (Yeong et al. 2024). In Brazil, barriers to timely and high-quality cancer care are well documented and include social vulnerability and pronounced inequalities in the geographic distribution of oncology resources (Domenico and Kalinke 2024).

In particular, there is a significant mismatch between the spatial distribution of cancer cases and the availability of radiotherapy equipment, with the highest levels of scarcity observed in the North and Northeast regions (Viani et al. 2022). Our results suggest that regions facing the greatest resource constraints did not experience reductions in radiotherapy waiting times comparable to those observed in the Center-South. In these contexts, the role of patient navigation appears to be limited by structural capacity constraints, highlighting the need to jointly consider geographical, epidemiological, logistical, and economic factors in radiotherapy planning and expansion (Fonseca et al. 2022, Hanna et al. 2022).

The relevance of navigation programs aimed at identifying and overcoming barriers to access has increasingly been recognized in Brazil. Several local initiatives have been implemented in recent years. In Amazonas, a state in the North region, a navigation service for 186 breast cancer patients achieved an 85% satisfaction rate (Lobo et al. 2025). In Minas Gerais, in the Southeast region, a pilot program involving 124 patients documented a 13-day reduction in the interval between biopsy results and referral for radiotherapy (Vieira et al. 2023). These experiences reinforce the potential of patient navigation to improve timely access to treatment (Bukowski et al. 2017) and reduce disparities along the oncology care continuum (Dixit et al. 2021).

## Conclusion

Our results indicate that short-term reductions in waiting times are accompanied by increased costs to the federal government, likely reflecting pent-up demand for procedures among patients who previously faced access barriers. In this context, the subsequent expansion of the National Patient Navigation Program to all cancer types is consistent with the observed reductions in waiting times and the increase in procedures.

However, the implementation of integrated care programs with navigation also presents significant challenges, particularly with respect to healthcare expenditures. Beyond higher spending on patient care, additional concerns relate to the financial and organizational resources required to train and allocate healthcare professionals—primarily nurses—with the specialized skills needed to act as patient navigators (Rodrigues et al. 2021). Future research should examine which population groups benefit most from PNPs, with particular attention to vulnerable populations and regional disparities. Further work could also assess the medium- and long-term fiscal implications of navigation programs, including the costs associated with training, staffing, and sustaining a specialized navigation workforce.

## Supplementary Material

czag105_Supplementary_Data

## Data Availability

The R code used in this study is publicly available at https://github.com/lanar-hub/Patient-navigation. The analytical dataset generated from the original data is available from the corresponding author upon reasonable request. Owing to the large size of the files, the datasets could not be included as supplementary material. The original data are publicly available for download from the DATASUS website (https://datasus.saude.gov.br/transferencia-de-arquivos/).

## Reflexivity statement

The authors include one woman and one man, spanning different levels of seniority. One author is an early career postdoctoral researcher, while the other is a full professor at the University of São Paulo. Both have experience in quantitative research, impact evaluation, and health policy, with a particular focus on the design, implementation, and evaluation of health policies in Brazil.
