# Supplementary material for: The Brazilian breast cancer patient navigation program: impact on waiting times and treatment costs
Source: Health Policy Plan. 2026 Aug 18;41(9):1568–79. doi: 10.1093/heapol/czag105 (PMC13678738; doi:10.1093/heapol/czag105)
Supplement: Supplementary file 1 [file czag105_supplementary_data.zip › 10. SUPPLEMENTARY_TABLE#3_ITS_GYNEC.docx]

Supplementary Table 3: Robustness check. Estimates and standard erros of interrupted time series (ITS) models for ginecological cancers.

|  | **Mean time** | | | **Cost** | | |
| --- | --- | --- | --- | --- | --- | --- |
|  | **Surgery** | **Chemotherapy** | **Radiotherapy** | **Surgery** | **Chemotherapy** | **Radiotherapy** |
| Intercept | 13.994***  (2.450) | 127.046***  (8.439) | 141.326***  (5.982) | 11.851***  (0.264) | 12.999***  (0.321) | 12.137***  (0.355) |
| Time | -0.060  (0.176) | -0.683  (0.642) | -0.061  (0.554) | 0.005  (0.020) | 0.006  (0.024) | 0.009  (0.026) |
| Post-intervention level | -0.139  (2.963) | -4.041  (11.652) | -1.921  (10.548) | 0.013  (0.426) | 0.045  (0.509) | -0.020  (0.550) |
| Post-intervention trend | -0.376  (0.233) | -0.536  (0.809) | -1.105  (0.745) | 0.008  (0.037) | 0.008  (0.044) | -0.005  (0.048) |
| Observations | 42 | 42 | 42 | 42 | 42 | 42 |

Note: The post-intervention trend coefficient captures the changes in the monthly slope of waiting time after program implementation compared to the pre-intervention period. In line with the SDID results, ITS estimates indicate that the intervention did not affect waiting times in any treatment modality. *** p < 0.01; ** p < 0.05; * p < 0.10.
