# Supplementary material for: The Brazilian breast cancer patient navigation program: impact on waiting times and treatment costs
Source: Health Policy Plan. 2026 Aug 18;41(9):1568–79. doi: 10.1093/heapol/czag105 (PMC13678738; doi:10.1093/heapol/czag105)
Supplement: Supplementary file 1 [file czag105_supplementary_data.zip › 11. SUPPLEMENTARY_TABLE#4_SDID_STAGING.docx]

|  | **Panel A:**  **Radiotherapy** |  | **Panel B:**  **Chemotherapy** |
| --- | --- | --- | --- |
|  |  |  |  |
|  | **SDID** |  | **SDID** |
| Advanced-stage | -0.064*** |  | 0.016 |
| SE | (0.013) |  | (0.005) |
| p-value | 0.000 |  | 0.002 |
| N | 20 |  | 20 |

Supplementary Table 4: Robustness check. SDID estimates for the share of advanced-stage (III–IV) cases.

Note: The table presents Synthetic Difference-in-Differences (SDID) estimates with standard errors per placebo, p-values, and sample sizes. *** p < 0.01; ** p < 0.05; * p < 0.10.
