# Supplementary material for: The Brazilian breast cancer patient navigation program: impact on waiting times and treatment costs
Source: Health Policy Plan. 2026 Aug 18;41(9):1568–79. doi: 10.1093/heapol/czag105 (PMC13678738; doi:10.1093/heapol/czag105)
Supplement: Supplementary file 1 [file czag105_supplementary_data.zip › 12. SUPPLEMENTARY_TABLE#5_PER_SUS - Copia.docx]

Supplementary Table 5a: Robustness check. Municipalities covered by the PER-SUS linear accelerator expansion

program and corresponding operating license dates.

| **Municipality** | **State** | **Date of operating license** |
| --- | --- | --- |
| Joaçaba | Santa Catarina (SC) | 23 May 2023 |
| Fortaleza | Ceará (CE) | 19 October 2023 |
| Santa Maria | Rio Grande do Sul (RS) | 10 January 2024 |
| Jacareí | São Paulo (SP) | 9 April 2024 |

Note: This table shows the municipalities with the operating license of accelerators as of January 2023.

Supplementary Table 5b: Robustness check for radiotherapy outcomes. SDID estimates excluding states that received PER-SUS linear accelerators during the post-intervention period

|  | **Panel A:**  **Mean time** |  | **Panel B:**  **Total costs** |
| --- | --- | --- | --- |
|  |  |  |  |
| SDID estimates | -7.205** |  | 0.047** |
| SE | (3.300) |  | (0.023) |
| p-value | 0.029 |  | 0.037 |
| N | 20 |  | 16 |

Note: The table presents Synthetic Difference-in-Differences (SDID) estimates with standard errors per placebo, p-values, and sample sizes.

*** p < 0.01; ** p < 0.05; * p < 0.10.
