# Supplementary material for: The Brazilian breast cancer patient navigation program: impact on waiting times and treatment costs
Source: Health Policy Plan. 2026 Aug 18;41(9):1568–79. doi: 10.1093/heapol/czag105 (PMC13678738; doi:10.1093/heapol/czag105)
Supplement: Supplementary file 1 [file czag105_supplementary_data.zip › 13. SUPPLEMENTARY_TABLE#6_C53.docx]

| Control unit | Radiotherapy (time) | Radiotherapy (cost) |
| --- | --- | --- |
| Vulva (C51) | 0.327 | 0.271 |
| Vagina (C52) | 0.327 | 0.246 |
| Cervix uteri (C53) | 0.000 | 0.000 |
| Corpus uteri (C54) | 0.346 | 0.277 |
| Uterus not otherwise specified (C55) | 0.000 | 0.000 |
| Ovary (C56) | 0.000 | 0.000 |
| Other and unspecified female genital organs (C57) | 0.000 | 0.000 |
| Placenta (C58) | 0.000 | 0.000 |
| Pre-treatment periods | 5 | 6 |

Supplementary Table 6a: Robustness check. Unit weights used in the SDID estimations excluding cervical cancer (C53).

Note: This table reports the unit weights used by the Synthetic Difference-in-Differences (SDID) estimator to construct the synthetic control group in each specification. Weights indicate the contribution of the control units to the synthetic comparison group and sum to one across control observations.

Supplementary Table 6b: Robustness check. SDID estimates excluding cervical cancer (C53).

|  | **Panel A:**  **Mean time** |  | **Panel B:**  **Total costs** |
| --- | --- | --- | --- |
|  |  |  |  |
| SDID estimates | -9.224*** |  | 0.132*** |
| SE | (0.001) |  | (0.046) |
| p-value | 0.000 |  | 0.008 |
| N | 15 |  | 24 |

Note: The table presents Synthetic Difference-in-Differences (SDID) estimates with standard errors per placebo, p-values, and sample sizes.

*** p < 0.01; ** p < 0.05; * p < 0.10.
