# Supplementary material for: The Brazilian breast cancer patient navigation program: impact on waiting times and treatment costs
Source: Health Policy Plan. 2026 Aug 18;41(9):1568–79. doi: 10.1093/heapol/czag105 (PMC13678738; doi:10.1093/heapol/czag105)
Supplement: Supplementary file 1 [file czag105_supplementary_data.zip › 7. SUPPLEMENTARY_TABLE#1_WEIGHTS.docx]

Supplementary Table 1: Unit weights used in the SDID estimations.

| Control unit | Surgery  (time) | Surgery  (cost) | Chemotherapy (time) | Chemotherapy (cost) | Radiotherapy (time) | Radiotherapy (cost) |
| --- | --- | --- | --- | --- | --- | --- |
| Vulva (C51) | 0.135 | 0.127 | 0.175 | 0.191 | 0.237 | 0.000 |
| Vagina (C52) | 0.120 | 0.123 | 0.000 | 0.000 | 0.247 | 0.000 |
| Cervix uteri (C53) | 0.159 | 0.133 | 0.180 | 0.216 | 0.256 | 0.865 |
| Corpus uteri (C54) | 0.151 | 0.134 | 0.172 | 0.225 | 0.260 | 0.135 |
| Uterus not otherwise specified (C55) | 0.141 | 0.120 | 0.000 | 0.000 | 0.000 | 0.000 |
| Ovary (C56) | 0.146 | 0.128 | 0.180 | 0.219 | 0.000 | 0.000 |
| Other and unspecified female genital organs (C57) | 0.150 | 0.127 | 0.120 | 0.149 | 0.000 | 0.000 |
| Placenta (C58) | 0.000 | 0.107 | 0.173 | 0.000 | 0.000 | 0.000 |
| Pre-treatment periods | 4 | 4 | 4 | 1 | 6 | 3 |

Note: This table reports the unit weights used by the Synthetic Difference-in-Differences (SDID) estimator to construct the synthetic control group in each specification. Weights indicate the contribution of the control units to the synthetic comparison group and sum to one across control observations.
