# Supplementary material for: The Brazilian breast cancer patient navigation program: impact on waiting times and treatment costs
Source: Health Policy Plan. 2026 Aug 18;41(9):1568–79. doi: 10.1093/heapol/czag105 (PMC13678738; doi:10.1093/heapol/czag105)
Supplement: Supplementary file 1 [file czag105_supplementary_data.zip › 9. SUPPLEMENTARY_TABLE#2_ITS_BREAST.docx]

Supplementary Table 2: Robustness check. Estimates and standard erros of interrupted time series (ITS) models for breast cancer.

| **Mean time** | | | | **Cost** | | |
| --- | --- | --- | --- | --- | --- | --- |
|  | **Surgery** | **Chemotherapy** | **Radiotherapy** | **Surgery** | **Chemotherapy** | **Radiotherapy** |
| Intercept | 67.373***  (2.267) | 118.635***  (2.505) | 180.165***  (4.299) | 15.115***  (0.019) | 17.612***  (0.009) | 15.908***  (0.024) |
| Time | -0.399***  (0.140) | -0.158  (0.151) | 0.849***  (0.290) | 0.012***  (0.001) | 0.006***  (0.001) | 0.011***  (0.002) |
| Post-intervention level | 8.479***  (2.211) | -2.907  (2.386) | 8.258  (5.335) | -0.084***  (0.030) | 0.046**  (0.022) | 0.075  (0.050) |
| Post-intervention trend | -1.852***  (0.240) | -1.219***  (0.195) | -5.044***  (0.513) | 0.002  (0.002) | 0.004**  (0.001) | -0.000  (0.004) |
| N | 42 | 42 | 42 | 42 | 42 | 42 |

Note: The post-intervention trend coefficient captures the changes in the monthly slope of waiting time after program implementation compared to the pre-intervention period. The models are estimated separately by treatment type, only for breast cancer patients. Consistent with the SDID results, the ITS estimates indicate a substantially stronger post-intervention decline in waiting times for radiotherapy compared to other treatment modalities. *** p < 0.01; ** p < 0.05; * p < 0.10.
